# Processing Methods of *Citrus* Medicinal and Edible Homologous Herbs: From Traditional Paozhi to Modern Technologies and Their Effects

**DOI:** 10.3390/foods15152691

**Published:** 2026-07-30

**Authors:** Jiangtao Liu, Yabo Li, Ying Liang, Asif Faryal, Yuxiao Wang, Yulong Bao, Lijun Zhu, Zhiyong Chen, Hongxun Tao, Yu Peng

**Affiliations:** 1 School of Food Science and Engineering, Jiangsu University, Zhenjiang 212013, China; 2 School of Pharmaceutical Sciences, Dalian University of Technology, Dalian 116024, China; 3 Key Laboratory of Food Processing Technology and Quality Control of Shandong Higher Education Institutes, College of Food Science and Engineering, Shandong Agricultural University, Taian 271018, China; 4 Shaanxi Academy of Traditional Chinese Medicine, Xi’an 710003, China; chenzhiyong0612@sina.com

**Keywords:** *Citrus* plants, medicinal and edible homologous herbs, processing technology, processing mechanism

## Abstract

*Citrus* plants are vital, globally distributed medicinal and edible homologous resources with a long application history in traditional Chinese medicine (TCM) and modern functional food industries. Representative *Citrus*-derived medicinal and edible herbs including *Citri reticulatae pericarpium* (*Chenpi*), *Citri grandis exocarpium* (*Huajuhong*), *Aurantii fructus immaturus* (*Zhishi*), *Aurantii fructus* (*Zhiqiao*), and *Citri reticulatae semen* (*Juhe*) possess rich bioactive components and diverse pharmacological activities, laying a solid material foundation for their dual food and medicinal applications. This review systematically summarizes the traditional empirical processing and modern innovative processing technologies of typical *Citrus* medicinal and edible homologous herbs, and elaborates the dynamic variations in core phytochemicals induced by different processing methods and parameters. Combined with modern phytochemistry and pharmacological research, the core chemical transformation mechanisms during processing, including hydrolysis, oxidation, polymerization and isomerization, are clarified, scientifically interpreting the TCM processing theory of “processing modulates herbal efficacy”. Meanwhile, the current research limitations of *Citrus* processing research have been analyzed. On this basis, future research directions are proposed, aiming to provide systematic theoretical support and practical technical guidance for the standardized processing, quality optimization, rational utilization and industrial upgrading of *Citrus* medicinal and edible homologous resources, and promote the cross-integrated development of TCM processing theory and modern food science and pharmacology.

## 1. Introduction

### 1.1. Overview of Citrus Genus

The *Citrus* genus, belonging to the *Rutaceae* family, encompasses more than 30 species and hundreds of cultivars, distributed pantropically across tropical and subtropical zones worldwide [1]. As one of the primary centers of origin for *Citrus* plants, China holds a leading global position in both citrus production and consumption, with a continuous cultivation history dating back over 4000 years. Beyond their popularity as fresh fruits, which are valued for their unique organoleptic qualities and high nutritional content, *Citrus* fruits also serve as vital raw materials in the food processing industry, supporting the production of fruit juices, jams, essential oils, and other derived products [2]. Notably, various botanical parts of *Citrus* species, including the pericarp, seeds, flowers, and leaves, have been widely used in traditional Chinese medicine (TCM) and ethnomedical practices for thousands of years. This long-standing application is attributed to their abundant phytochemical components and diverse bioactive properties. *Citrus* plants are rich in a wide range of bioactive compounds, such as flavonoids, limonoids, essential oils, polysaccharides, phenolic acids, coumarins, and alkaloids. These phytochemical constituents have been shown to exhibit diverse pharmacological activities, including antioxidant, anti-inflammatory, antitumor, and cardioprotective effects—findings that provide a scientific foundation for classifying *Citrus* species as medicinal and edible homologous resources [3]. With the growing global focus on functional foods and natural product-based therapies, the medicinal and nutritional potential of *Citrus* genus plants has attracted considerable attention from both academic researchers and industrial professionals. As a result, in-depth studies on processing techniques and the mechanisms underlying their bioactivities will become key research priorities at the crossroads of food science and TCM pharmacology.

### 1.2. Connotation and Advantages of Medicinal–Edible Homology

Medicinal and edible homologous herbs (MEHHs) refer to natural materials that possess both edible safety and notable pharmacological activities. Under the guidance of evidence-based medicine and traditional health-preserving theories, they can be consumed as daily foods while exerting preventive and therapeutic effects against certain diseases [4]. In China, the National Health Commission has successively released official lists of medicinal and edible homologous items, among which several *Citrus*-derived materials are formally included. These can be categorized according to their plant parts: (1) pericarps, including *Citri reticulatae pericarpium* (*Chenpi*, 陈皮) and *Citri grandis exocarpium* (*Huajuhong*, 化橘红); (2) immature and unripe fruits, namely *Aurantii fructus immaturus* (*Zhishi*, 枳实) and *Aurantii fructus* (*Zhiqiao*, 枳壳); and (3) seeds, represented by *Citri reticulatae semen* (*Juhe*, 橘核) [3]. Their official recognition has further confirmed their reliable safety and dual value as both medicinal and nutritional resources. The concept of medicinal–edible homology is deeply rooted in traditional Chinese medicine (TCM), reflecting the long-standing practice of integrating medical treatment and dietary care in the philosophy of “treating illness through food” [4]. *Citrus-*derived MEHHs exhibit prominent strengths including wide resource distribution, low toxicity, and good biocompatibility, making them highly consistent with modern preventive healthcare demands. Over recent decades, with the rapid development of natural product chemistry and pharmacological research, *Citrus* MEHHs have been increasingly applied in functional foods, dietary supplements, and pharmaceutical preparations, revealing considerable market prospects in the global health industry.

### 1.3. Properties and Efficacy of Typical Citrus Herbs

**Figure 1:**
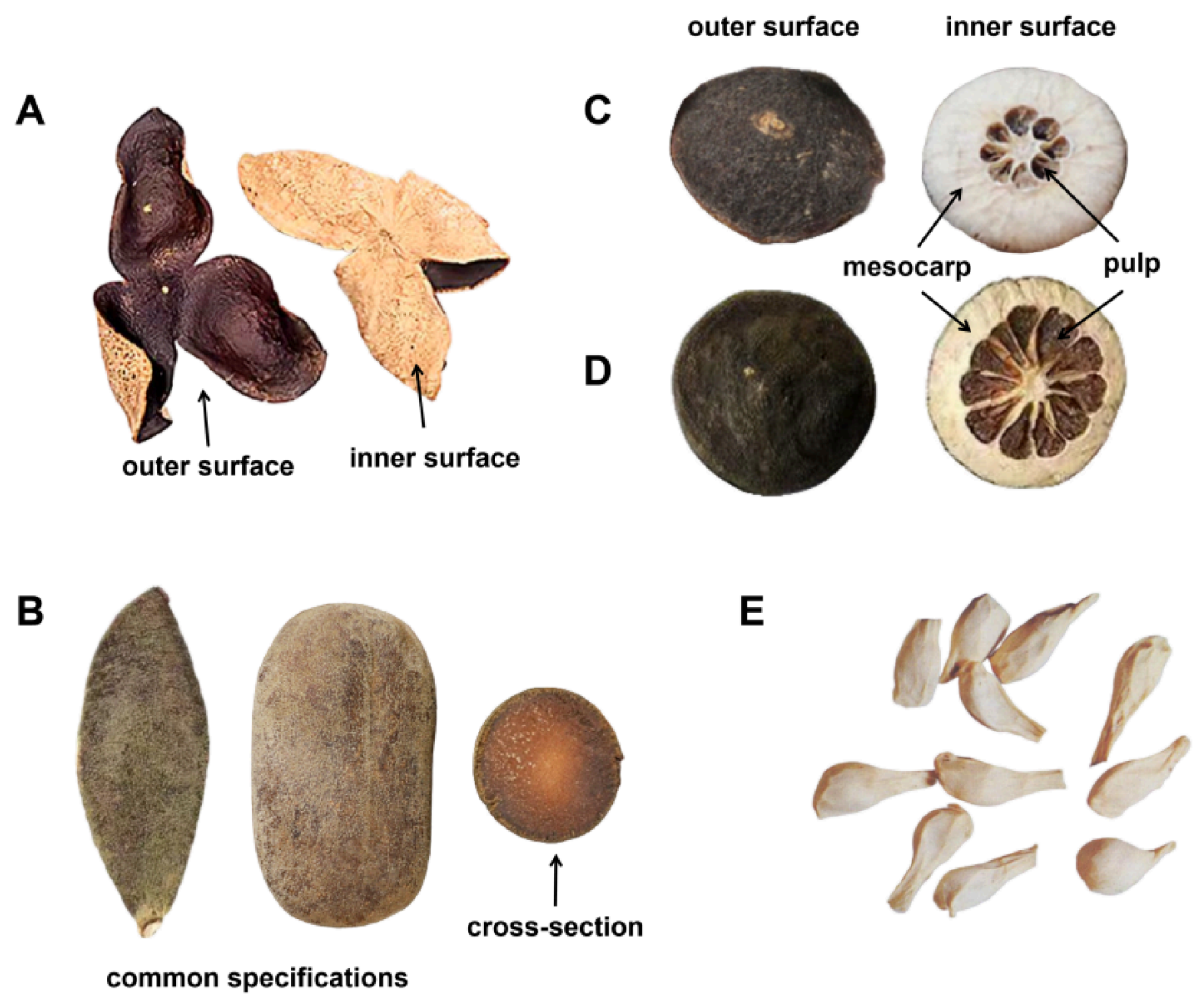
The main traditional Chinese medicinal materials of the *Citrus* genus ((**A**) *Chenpi*; (**B**) *Huajuhong*; (**C**) *Zhishi*; (**D**) *Zhiqiao*; (**E**) *Juhe*).

*Citri Reticulatae Pericarpium* (*Chenpi*, Figure 1A): As the dried pericarp of *Citrus reticulata* Blanco and its cultivars, *Chenpi* is one of the most commonly used medicinal–edible homologous herbs in traditional Chinese medicine (TCM). According to the Chinese Pharmacopoeia, apart from *Citrus reticulata*, the main cultivated varieties of *Chenpi* include *Citrus reticulata* ‘Chachi’ (*Guangchenpi*), *Citrus reticulata* ‘Dahongpao’, *Citrus reticulata* ‘Unshiu’ (Satsuma mandarin), and *Citrus reticulata* ‘Tangerina’ (*Fuju*) [5]. It is characterized by a warm nature, a pungent and bitter taste, and it targets the spleen and lung meridians. Its therapeutic effects include regulating *qi* (the vital life energy that maintains bodily health and balance in TCM theory), invigorating the spleen, eliminating dampness, and resolving phlegm. Clinically, it is widely used for treating spleen–stomach *qi* stagnation, abdominal distension, poor appetite, vomiting, diarrhea, and phlegm–damp cough [6].

*Citri Grandis Exocarpium* (*Huajuhong*, Figure 1B): Originating from the dried outer peel of *Citrus grandis* (L.) Osbeck var. tomentosa Hort. or *Citrus grandis* (L.) Osbeck, *Huajuhong* is a geo-authentic medicinal–edible resource unique to Guangdong and Guangxi provinces in China [5]. It has a warm nature, a pungent and bitter taste, and acts on the lung and stomach meridians, exerting antitussive, expectorant, *qi-*regulating, and distension-relieving effects. It is particularly effective in alleviating productive cough, asthma, thoracic distension, and abdominal fullness. Phytochemical studies have identified naringin as its major bioactive component, which is responsible for its cough-suppressing and phlegm-resolving properties [7].

*Aurantii Fructus Immaturus* (*Zhishi*, Figure 1C) is mainly cultivated in Jiangxi, Hubei, and Sichuan provinces of China. Its dried young fruits are harvested from May to June, when the fruit diameter reaches 2~3 cm, and then processed by air-drying or stir-frying. In contrast, *Aurantii Fructus* (*Zhiqiao*, Figure 1D) is collected from July to August when the fruits are still unripe, followed by processing methods such as drying, stir-frying, or bran-frying. Both *Zhishi* and *Zhiqiao* are rich in flavonoids (e.g., naringin, hesperidin) and limonoids, which serve as their primary bioactive components [8]. Notably, processing techniques significantly alter their phytochemical compositions and therapeutic effects, making processing a critical step for their clinical application.

*Citri Reticulatae Semen* (*Juhe*, Figure 1E): The dried seeds of *Citrus reticulata* Blanco and its cultivars (*Citrus reticulata* ‘*Dahongpao*’ and *Citrus reticulata* ‘*Tangerina*’) also serve as an important medicinal–edible homologous herb [5]. It has a warm nature, a pungent and bitter taste, and acts on the liver and kidney meridians, with therapeutic effects including *qi* regulation, pain relief, mass resolution, and liver soothing. Clinically, it is indicated for liver *qi* stagnation, hypochondriac pain, hernia pain, and breast distension. Modern processing methods, such as stir-frying and salt-processing, are commonly used to enhance its bioactivity and improve oral bioavailability [9].

Other relevant herbs: In addition to the four main herbs mentioned above, other parts of *Citrus* plants also have significant medicinal and nutritional value. For instance, the peel of *Citrus limon* (L.) Burm. f. (lemon peel) exhibits antioxidant, antibacterial, and lipid-lowering activities [10]. The peel of *Citrus paradisi* Macfad. (grapefruit peel) is rich in flavonoids and essential oils, which have been shown to possess anti-inflammatory and antitumor properties [11]. Meanwhile, the leaves of *Citrus reticulata* (*Juye*) exhibit significant antioxidant and anti-inflammatory activities, making them suitable for processing into functional teas and nutraceutical products [12]. A comparison of traditional processing methods for major *Citrus* MEHHs is shown in Table 1.

### 1.4. Research Progress, Deficiencies and Significance

In recent years, researchers have conducted extensive studies on the processing technologies and bioactive properties of *Citrus* MEHHs. Regarding processing methodologies, traditional techniques such as aging, sun-drying, and stir-frying have been comprehensively characterized through systematic research. Meanwhile, modern processing technologies, including low-temperature drying, vacuum drying, microwave drying, and freeze-drying, have been increasingly employed in practical production. These modern approaches not only improve processing efficiency but also enhance the overall quality of *Citrus* herb products, overcoming the limitations of traditional processing methods.

In terms of bioactivities, multiple pharmacological effects of *Citrus* herbs, such as antioxidant, anti-inflammatory, and gastrointestinal regulatory activities, have been verified using various in vitro and in vivo experimental models. Additionally, preliminary studies have clarified the main bioactive components and their potential mechanisms of action, laying a foundation for further in-depth research. However, current research on *Citrus* MEHHs still has several prominent limitations:

(1) Processing protocols for *Citrus* MEHHs lack standardization, and there are no unified quality control criteria. This inconsistency results in significant variations in product quality and therapeutic efficacy among different regions and manufacturers, impeding the industrialization and standardized development of these herbs.
(2) The molecular mechanisms underlying processing-induced changes in phytochemical profiles and bioactivities remain incompletely elucidated. In particular, the interactions between different components and the modulation of signaling pathways during processing have not been fully explored, restricting the optimization of processing technologies.
(3) Clinical evidence supporting the bioactivities of *Citrus* MEHHs is insufficient. Most existing studies are confined to in vitro experiments and pre-clinical animal models, and the therapeutic effects and safety of these herbs in humans still need to be validated through well-designed clinical trials.
(4) The bioavailability of certain active components, such as flavonoid glycosides, is relatively low. This drawback limits their effective translation into functional foods, nutraceuticals, and pharmaceutical formulations, reducing their practical application value.

The purpose of this review is to systematically summarize the current research progress on the processing technologies and changes in phytochemical profiles of *Citrus* MEHHs, including *Chenpi*, *Huajuhong*, *Juhe*, *Zhishi*, and *Zhiqiao*. Specifically, this work aims to clarify the tripartite relationship between processing techniques, chemical composition, and bioactivity; elaborate on the underlying processing mechanisms; and identify the existing challenges and future research priorities.

## 2. Traditional and Modern Processing Technologies of Typical *Citrus* MEHHs

The processing of *Citrus* MEHHs mainly falls into two categories: traditional processing (*paozhi*) and modern processing. Guided by traditional Chinese medicine theories, *paozhi* refers to the standardized processing system for crude medicinal materials, covering sorting, cutting, water/fire processing, aging and storage. It includes rutaceous herb-specific techniques like bran stir-frying, salt stir-frying, charring and low-temperature aging to reduce toxicity, regulate efficacy and stabilize quality, distinct from routine natural product extraction [5]. Specific processing techniques are tailored to the unique biological characteristics of different *Citrus* herbs, ensuring that the processing methods align with the herbs’ inherent properties. Beyond optimizing efficacy and reducing toxicity, this integrated processing system also improves sensory qualities, maintains chemical stability, and extends storage life by systematically regulating the biochemical composition of the herbs [13]. This underscores the irreplaceable role of processing in unlocking the full medicinal and nutritional value of *Citrus* MEHHs.

### 2.1. Processing of Citri reticulatae pericarpium (Chenpi)

#### 2.1.1. Traditional Processing Methods

The traditional processing of *Citri reticulatae pericarpium* (*Chenpi*) comprises four sequential steps: harvesting, peeling, drying, and aging. It is characterized by time-honored standardized procedures and accumulated empirical experience.

For optimal *Chenpi* production, *Citrus* fruits are harvested between October and December, during their physiological ripening period. This is when secondary metabolites, the key bioactive components, reach their highest accumulation levels. After harvesting, the fruits are thoroughly cleaned. Subsequently, manual or mechanical peeling is carried out to obtain intact pericarp halves, with the navel kept intact to preserve quality. The peeled pericarp is then sun-dried in a well-ventilated, sun-exposed area until its moisture content is precisely controlled at 12~15%, resulting in an intermediate product known as “fresh peel” [14].

Notably, fresh peel requires controlled aging to enhance its therapeutic efficacy, making aging the rate-limiting step in traditional *Chenpi* processing. Aging is a complex biotransformation process involving physical, chemical, and microbial-driven changes, with a minimum required duration of 3 years [5]. During this period, the pericarp is stored in climate-controlled warehouses that maintain low humidity, adequate ventilation, and cool temperatures. Regular turning is also performed to prevent microbial contamination and insect infestation. Traditional aging methods focus on “natural fermentation”, where fluctuations in ambient temperature, humidity gradients, and native microbial communities work synergistically to drive phytochemical transformations [15]. This process promotes the degradation of bitter-tasting compounds, the accumulation of bioactive phenolics, and the formation of unique volatile profiles, all of which enhance both the therapeutic efficacy and organoleptic qualities of *Chenpi* [16].

*Chenpi*’s well-documented “the older, the better” quality attribute is closely linked to the progressive enrichment of phenolic acids during the aging process, which are widely recognized as reliable indicators of storage quality [17]. During aging, dynamic transformations occur in bioactive components, flavor metabolites, and microbial communities. Existing research confirms that oven-drying at 30 °C prior to aging elevates microbial diversity, laying a foundation for the colonization and metabolic transformation of functional microbiota during subsequent aging. Meanwhile, Aspergillus was identified as the dominant fungal genus in the late stage of one-year aging (270–360 days), and its relative abundance was positively correlated with the accumulation of flavonoids. This indicates that drying regimens can profoundly remodel microbial community structures and further determine the overall quality of Citri Reticulatae Pericarpium. These outcomes also provide theoretical support for optimizing aging protocols, establishing quality control systems, and developing scientific warehousing strategies [18].

Beyond the primary aging and drying steps, *Chenpi* also undergoes several specialized processing variants, including carbonization (*Chenpi tan*, 陈皮炭), earth-frying (*tu chao Chenpi*, 土炒陈皮), bran-frying (*mai fu chao Chenpi*, 麸炒陈皮), and salt-frying (*yan chao Chenpi*, 盐炙陈皮) [14]. Carbonized *Chenpi* is prepared by stir-frying at 200~220 °C until the surface is charred while maintaining structural integrity. No prior studies have systematically explored how heating duration and heat transfer mechanisms mediate the balance between surface carbonization and intact internal tissue structure, and key efficiency parameters of this treatment are yet to be clearly quantified. This process increases tannin content, enhancing hemostatic and anti-diarrheal effects, and is clinically indicated for gastrointestinal hemorrhage and chronic enteritis. Earth-frying with loess (Terra Flava Usta) at 120~140 °C leads to the surface adsorption of mineral components, which strengthens the herb’s spleen-invigorating and dampness-resolving properties. Bran-frying with wheat bran at 100~110 °C reduces volatile oil content by 30~40%, alleviating its pungent taste and improving palatability [15]. Salt-frying with a 2% NaCl solution enables ionic modification of flavonoid glycosides, enhancing its affinity for targeting the renal meridian and reinforcing its *qi-*regulating and analgesic effects, particularly in the management of dysmenorrhea [19]. At present, this conclusion is merely inferred from experimental observations. Beyond the ionic modification pathway, more complicated underlying mechanisms are involved, which remain to be further elucidated.

#### 2.1.2. Modern Processing Methods

With the rapid advancement of food science and technology, modern processing technologies are increasingly employed in *Chenpi* production. These technologies offer distinct advantages, such as high efficiency, stable performance, and controllable quality, which address the limitations of traditional processing methods.

Low-temperature drying is one of the most commonly used modern techniques. Fresh peel is dried in a low-temperature oven set at 40~60 °C. This effectively avoids the loss of volatile components and bioactive substances that typically occur under high-temperature conditions [20]. This gentle drying approach ensures the retention of key phytochemicals, thereby preserving *Chenpi*’s medicinal and nutritional value. Vacuum drying is another crucial modern processing method. By conducting drying under vacuum conditions, this technology further reduces the required drying temperature and shortens the processing time, while better retaining the inherent chemical composition and therapeutic efficacy of *Chenpi*. Compared to traditional sun-drying, vacuum drying minimizes the oxidative degradation of active components such as flavonoids and essential oils, contributing to more consistent product quality [21]. Infrared drying, which relies on infrared radiation for heating, has the characteristics of rapid heating, uniform drying, and high processing efficiency. This method not only accelerates the drying process but also improves the overall quality of *Chenpi* by ensuring uniform moisture distribution and reducing the risk of microbial contamination during processing [22].

In addition to drying technologies, modern aging techniques have also been developed to optimize *Chenpi* maturation. Controlled temperature and humidity aging is a typical example. This approach precisely regulates the storage environment, maintaining a temperature of 20~25 °C and a humidity of 60~70% to simulate the natural aging process [23]. It not only significantly shortens the aging period but also ensures consistent quality across batches of *Chenpi*, addressing the variability issues associated with traditional natural aging.

Furthermore, some researchers have explored microbial fermentation to accelerate *Chenpi* aging. By inoculating specific microorganisms (e.g., lactic acid bacteria and yeast) onto the peel, this method promotes the biotransformation of phytochemical components, enhances the formation of bioactive substances, and ultimately improves *Chenpi*’s therapeutic efficacy [24]. This innovative approach combines modern microbiology with traditional processing, providing a new pathway for efficient *Chenpi* production.

### 2.2. Processing of Citri grandis exocarpium (Huajuhong)

#### 2.2.1. Traditional Processing Methods

The traditional processing of *Citri grandis exocarpium* (*Huajuhong*) consists of four sequential steps: harvesting, peeling, drying, and post-processing. Each step is closely linked to the herb’s geographical origin and therapeutic efficacy. Optimal harvesting takes place between July and August, when the fruits are still immature (with a diameter of 5~7 cm). This stage is critical, as it maximizes the accumulation of bioactive flavonoids, the key components underlying *Huajuhong*’s medicinal value. After harvesting, the fruits are thoroughly cleaned. Then, the outer pericarp is mechanically separated, with the inner peel and pulp completely removed to ensure product purity. The outer pericarp is then cut into standardized strips or pieces before the drying process begins [5].

Two primary thermal processing methods dominate traditional *Huajuhong* drying: fire roasting and charcoal simmering. Fire roasting involves controlled heating over a low-intensity flame until the pericarp surface turns a characteristic brownish–yellow and aromatic compounds are volatilized. In contrast, charcoal simmering uses indirect heating in a closed charcoal-fired vessel, continuing until the pericarp is fully dried and its unique aroma is fully developed. Both techniques facilitate moisture removal, enrich volatile compounds, and stabilize phytochemical components. These effects have been achieved through heat-induced transformations of polymethoxyflavones and essential oils, which are critical for maintaining *Huajuhong*’s efficacy [25].

Solar drying is an alternative traditional approach, relying on solar radiation to reduce the pericarp’s moisture content to 10~12%. However, this method has notable limitations: it is highly weather-dependent, requires an extended processing period (3~5 days), and may lead to losses of thermally sensitive volatile components (e.g., limonene, γ-terpinene). Additionally, it carries an increased risk of microbial contamination due to the prolonged exposure to ambient conditions [26]. For these reasons, thermal processing methods (fire roasting and charcoal simmering) remain the predominant traditional techniques for *Huajuhong* processing, as they offer superior process control and better preserve the herb’s phytochemical profile and therapeutic efficacy.

#### 2.2.2. Modern Processing Methods

Modern processing technologies for *Huajuhong* are primarily focused on improving drying efficiency, enhancing product quality, and minimizing the loss of bioactive components, thereby addressing the inherent limitations of traditional processing methods.

Hot air drying is one of the most widely used modern techniques. It employs controlled hot air at 50~60 °C to dehydrate the outer pericarp. Its key advantages include rapid moisture removal, uniform drying kinetics, and precise temperature control, ensuring consistent product quality across batches [27]. Microwave drying relies on electromagnetic radiation to generate volumetric heating within the pericarp tissue, enabling rapid moisture evaporation while effectively preserving key phytochemicals (e.g., naringin) and volatile aromatic compounds [28]. Compared to conventional drying methods, this technology achieves dehydration rates 3~5 times faster, while maintaining the cellular integrity of the pericarp, which is critical for retaining the herb’s medicinal and sensory properties. Freeze-drying is an advanced dehydration technology that involves cryogenic freezing at −40 to −50 °C, followed by sublimation under vacuum conditions. This gentle processing approach maximizes the retention of thermolabile constituents and bioactive efficacy. However, its high energy consumption restricts its application to premium-grade *Huajuhong* products [29]. Recent research has shown that freeze-dried *Huajuhong* samples retain 18~23% more flavonoids compared to those processed via traditional thermal methods, highlighting its superiority in preserving bioactive components [30].

Innovative hybrid processing technologies have also been developed in recent years. These integrated systems synergize the advantages of multiple drying methods. For instance, a novel two-stage sequential drying strategy, integrating hot air drying (HAD)-intermittent tempered hot air drying (ITHC) with radio frequency (RF) drying, has been developed to address the challenges of prolonged drying duration and degradation of heat-sensitive bioactive compounds in large-sized *Huajuhong*. The optimal moisture content transition point has been identified as 40%, and critical process parameters for the two RF hybrid drying configurations have been systematically optimized. The results demonstrate that the HAD-ITHC-RF-HA mode has exhibited the most comprehensive performance, reducing drying time by 30.01~37.15% while increasing flavonoid content by 5.92~9.41%, thus achieving an optimal balance between drying efficiency and product quality [31]. This study breaks traditional limitations, and improves *Huajuhong* drying efficiency and quality; HAD-ITHC-RF-HA fits large-scale production, supporting standardized processing of its medicine-food products.

### 2.3. Processing of Aurantii fructus immaturus (Zhishi) and Aurantii fructus (Zhiqiao)

#### 2.3.1. Traditional Processing Methods

The traditional processing of *Aurantii fructus immaturus* (*Zhishi*) and *Aurantii fructus* (*Zhiqiao*) mainly relies on dehydration and thermal processing techniques. Scientific research has confirmed that these processing methods not only enhance the stability of bioactive components and optimize therapeutic efficacy but also reduce the gastrointestinal irritation inherent in raw materials, laying a foundation for their safe clinical application and daily health use.

For *Zhishi*, the standardized traditional processing procedure follows a clear workflow: harvesting immature fruits (with a diameter of 2~3 cm) → ultrasonic cleaning → longitudinal sectioning (bisected or quadrisected, with optional seed removal) → dehydration via solar drying or shade drying until the moisture content is reduced to 10~12% [5]. Two primary stir-frying methods are commonly used for further processing: bran-frying (麸炒) and honey-frying (蜜炙). Bran-fried *Zhishi* is prepared by stir-frying with wheat bran at 120~140 °C until the surface turns yellowish–brown. Eleven differential components have been screened from *Zhishi* and bran-fried *Zhishi*; compared with *Zhishi*, the relative contents of most chemical components in bran-fried *Zhishi* increase (including adenosine, narirutin, hesperidin, nobiletin, 5,7,8,4′-tetramethoxyflavone, and demethylnobiletin). Only syringetin-3-*O*-glucoside shows reduced content with a variation index of 0.89 [32]. Honey-fried *Zhishi* involves stir-frying with 15% (*w*/*w*) honey syrup until the surface presents a glossy amber appearance. Volatile oil analysis revealed that, compared with raw *Zhishi*, 52 novel compounds have been detected in bran-fried *Zhishi*, and 26 new compounds have been identified in honey-bran-fried *Zhishi*. Concomitantly, the contents of certain compounds either increase or decrease. These findings suggest that the thermal effects during processing, as well as potential interactions with excipients, may be key factors contributing to alterations in the content and profiles of volatile oil components [33]. Raw *Zhishi* is predominantly utilized for *qi* promotion and distension relief. Following bran-frying, its therapeutic effects become milder, with a tendency for *qi* regulation and spleen invigoration. Honey serves as a commonly employed liquid excipient in traditional Chinese medicine processing. Honey-fried herbal medicines are known for enhancing therapeutic effects such as cough relief, lung moistening, spleen tonification, and *qi* replenishment.

For *Zhiqiao*, the traditional processing workflow differs slightly: harvesting semi-mature fruits → ultrasonic cleaning → transverse sectioning into 3~5 mm slices or cuboids → primary dehydration (either solar drying or low-temperature baking at 50~60 °C) → secondary thermal processing [5]. In addition to being processed with bran and honey, *Zhiqiao* is also fermented in the Guangdong–Hong Kong region. There are significant differences in the contents of naringin and marmesin before and after bran-frying: the naringin content increases while the marmesin content decreases, confirming the component difference between raw and processed products [34]. As a fat-soluble component, the decrease in marmesin may be related to bran-frying reducing the volatile oil content of *Zhiqiao*, reducing irritating components, so as to achieve “relieving dryness and harmonizing the stomach”. A study on Zhangbang honey-bran-fried *Zhiqiao* focused on its characteristic processing technology. The flavonoid components changed with the thermal decomposition reaction, and the honey bran could buffer the thermal effect to achieve the effect of reducing dryness and enhancing efficacy. Meanwhile, the synergistic changes between terpenoids and flavonoids provided a scientific basis for the standardized processing of *Zhiqiao* [35]. On the other hand, fermentation processing can deglycosylate flavonoid glycosides (neeriocitrin, hesperidin, neohesperidin) in *Zhiqiao* into monoglycosides or aglycones, reducing polarity and increasing fat solubility, which is conducive to small intestinal absorption and efficacy exertion. Studies have also shown that monoglycosides and aglycones produced by hesperidin hydrolysis can improve its bioavailability, suggesting that after fermentation processing, *Zhiqiao* may enhance clinical efficacy by increasing the bioavailability of active components [36].

#### 2.3.2. Modern Processing Methods

Modern processing technologies for *Zhishi* and *Zhiqiao* are focused on three core goals: improving processing efficiency, standardizing quality control, and maximizing the retention of bioactive components. A study compared four drying methods (hot air, vacuum, microwave, infrared) with vacuum freeze-drying as the control, analyzing their effects on the drying kinetics, physical properties, and quality of *Aurantii Fructus*. A total of 64 volatile compounds were detected, with 20 being common across the methods. Vacuum drying yielded the highest terpenoid content, while vacuum freeze-drying retained the most linalool. Microwave drying exhibited optimal retention of total phenols and flavonoids, along with the best antioxidant capacity and color preservation. Overall, microwave drying was identified as the most suitable method for *Aurantii Fructus*, effectively improving its comprehensive quality [37].

Another study aimed to investigate the effects of drying methods on the appearance and chemical components of *Aurantii Fructus*, providing a scientific basis for selecting suitable drying and processing methods. Fresh-cut slice drying yielded a fast drying rate, low moisture content, and significantly higher contents of extract, naringin, and neohesperidin than other methods. Post-drying cutting and post-sun-drying showed no significant difference in appearance and internal quality, except for the epidermal color (greenish–brown for drying, brown for sun-drying). After storage, the epidermal color of *Aurantii Fructus* darkened, the moisture content increased, the contents of extract and volatile components decreased, while the naringin content increased and the neohesperidin content decreased. Comprehensively considering quality, drying rate, and economy, fresh-cut slice drying was identified as the most suitable for *Aurantii Fructus* processing [38].

### 2.4. Processing of Citri reticulatae semen (Juhe)

#### 2.4.1. Traditional Processing Methods

The traditional processing of *Citri reticulatae semen* (*Juhe*) primarily comprises cleaning, dehydration, and thermal processing. Although these procedures appear simple, they exert significant regulatory effects on the herb’s pharmacological properties, laying a foundation for its safe and effective application.

After harvesting, *Citrus* seeds are mechanically separated from the pulp, ultrasonically cleaned to remove impurities, and then sun-dried or air-dried until the moisture content is reduced to 10~12%, resulting in the crude drug material [39]. Raw *Juhe* has a strong pungent taste and low bioavailability due to its intact cell wall structure; thus, it requires further processing via stir-frying (炒制) to enhance therapeutic efficacy and improve the bioaccessibility of active components.

A study investigated the correlation between color parameters and chemical components, as well as the transformation mechanism of key components during the salt-frying and stir-frying processes of *Juhe*. During processing, color parameters show significant correlations with nomilin and obacunone contents, while limonin content remains relatively stable. When heated at 200~250 °C for 5~60 min, nomilin is significantly transformed into obacunone. In vitro anti-inflammatory assays demonstrated that high concentrations of nomilin and all tested concentrations of obacunone can significantly inhibit nitric oxide (NO) release from LPS-induced RAW264.7 cells (*p* < 0.01), with obacunone exhibiting stronger inhibitory effects [40].

Traditional Chinese medicine theory holds that salt-processed *Juhe* enhances the effects in treating hernia and breast swelling and pain; modern studies also reveal that salt-processed *Juhe* has stronger analgesic and anti-inflammatory activities than raw *Juhe*. Our investigations confirm that salt-processed *Juhe* is the main form used clinically. However, one study has shown significant differences in limonin and nomilin contents among salt-processed *Juhe* from different sources and batches. After processing, both limonin and nomilin contents decrease to varying degrees; the total contents of limonin and nomilin in salt-processed and stir-fried products are only 72.5% and 80% of those in raw products, respectively, while the ratio of limonin to nomilin remains relatively stable [41].

In addition, a screening study on the scavenging rate of nitrite and the blocking rate of nitrosamine synthesis revealed that both raw *Juhe* and salt-processed *Juhe* extracts exhibit strong nitrite scavenging effects. The nitrite scavenging rate increases after processing. Raw *Juhe* itself shows no blocking effect on nitrosamine, but exhibits a certain blocking effect after processing [42]. The aforementioned research cases lay the foundation for the scientific nature of *Juhe* processing, reflecting the wisdom of traditional Chinese medicine processing.

#### 2.4.2. Modern Processing Methods

Although research on modern processing of *Juhe* remains relatively limited to date, based on the characteristics of its active components and functional efficacy, and by referencing processing strategies for similar medicinal materials, the primary objectives of processing strategies should be as follows: improving processing efficiency, standardizing quality parameters, and enhancing the therapeutic bioavailability of active components.

The traditional and modern processing methods for different *Citrus* MEHHs have been compared and are summarized in Table 2. Rooted in long-term practical experience, traditional processing mainly adopts natural sun-drying, fire roasting, natural aging and auxiliary material stir-frying. It is constrained by long production cycles, environmental interference and inconsistent batch quality, while distinct harvesting schedules and exclusive processing techniques have been developed for individual *Citrus* MEHHs. In contrast, modern processing integrates advanced technologies such as diversified drying methods, controlled aging, microbial fermentation and combined innovative processes. It features high production efficiency, precise parameter control, high retention of active ingredients and superior batch-to-batch uniformity, effectively overcoming the defects of conventional techniques. Targeted technical strategies have also been established in accordance with the characteristics of different raw materials, laying a solid foundation for the standardization, large-scale production and quality improvement of *Citrus* MEHHs.

### 2.5. General Processing Principles

The processing of *Citrus* medicinal and edible homologous herbs adheres to the core principles of traditional Chinese medicine (TCM) processing. According to the Pharmacopoeia of the People’s Republic of China (2025 Edition), these principles are systematically classified into four categories: efficacy enhancement, toxicity attenuation, palatability improvement, and component stabilization [5]. *Citrus* peel is rich in functional ingredients, including essential oils (0.6~1%), fibers, phenols (0.67~19.62 g/100 g db), and vitamin C (0.109~1.150 g/100 g db). Its main phenolic compounds are flavanones such as hesperidin and neohesperidin, as well as polymethoxylated flavones including sinensetin and nobiletin. Their antioxidant activity allows for applications in food, cosmetics, and pharmaceuticals. Phenolics are sensitive to processing conditions and vulnerable to juice extraction, and thermal and non-thermal treatments. Both conventional and innovative processing methods can alter their content. Clarifying the effects of processing helps optimize techniques to maximally retain antioxidants in *Citrus* products [43].

Toxicity attenuation mainly targets the irritant components in *Citrus* herbs. For example, thermal modification can reduce the gastrointestinal irritation caused by limonin. Stir-frying of *Juhe*, for instance, decreases pungency by 40~50% through the partial degradation of volatile terpenoids, minimizing adverse effects while retaining therapeutic value [44]. Palatability improvement focuses on optimizing sensory properties, which is a key factor for functional food applications. This is achieved by reducing bitter compounds (e.g., naringin) and enhancing aromatic profiles through controlled Maillard reactions during processing, making *Citrus* herbs more acceptable for daily consumption and functional food formulation [45].

The processing efficiency and quality of *Citrus* herbs are governed by four critical variables: temperature, duration, humidity, and processing adjuvants. These factors collectively regulate the kinetic parameters of phytochemical transformation and determine the final quality attributes of the processed products. Temperature serves as a key kinetic regulator, directly affecting reaction rates and the retention of thermolabile components. Stir-frying *Chachi* mandarin peel at 110 ± 10 °C for 25 min alters its appearance and microstructure, improves sensory quality, lowers moisture, and contains no aflatoxin, while increasing total flavonoids. Eleven common chromatographic peaks exist between raw and stir-fried samples, seven of which are identified. Stir-fried peel exhibits enhanced antioxidant and α-glucosidase inhibitory (hypoglycemic) activities. Tangeretin correlates with antioxidant capacity and nobiletin with hypoglycemic potency, with overlapping and distinct bioactive components linking the two functions. This simple and practical stir-frying technique improves product quality and bioactivities, supporting its industrial application and providing technical evidence for the industrial conversion of this natural plant material [46]. Pressurized hydrothermal processing (subcritical water treatment) affects pectin hydrolysis. Such processing alters pectin’s molecular size and reducing-end content, with hydrolysis following zero-order reaction kinetics. The established empirical kinetic model, validated with a relative average bias of 9.42%, reveals that kinetic constants are temperature- and pressure-dependent and enables prediction of pectin’s molecular size reduction via reducing-end content at a given reaction time. Being clean and efficient, subcritical water-mediated hydrothermal treatment is a prospective method for manufacturing pectin derivatives and guides parameter optimization for practical hydrolysis processes [47].

## 3. Effects of Processing on Phytochemical Composition of *Citrus* MEHHs

Processing induces significant perturbations in the chemical profiles of *Citrus* medicinal and edible homologous herbs. These perturbations encompass quantitative variations, structural modifications, and solubility changes in their primary bioactive constituents, including flavonoids (e.g., hesperidin, naringin), limonoids (limonin, nomilin), volatile oils (D-limonene, linalool), and acidic polysaccharides. Among these components, flavonoids stand out as the predominant ones, exerting diverse pharmacological activities such as antioxidant, anti-inflammatory, and antitumor effects by regulating cellular signaling pathways. Representative compounds include hesperidin (7-*O*-rutinoside), naringin (7-*O*-neohesperidoside), their aglycones naringenin and hesperetin, quercetin, and rutin (3-*O*-rutinoside). Processing-driven transformations of these flavonoids mainly occur via glycosidic bond hydrolysis, oxidative coupling, and polymerization reactions to modify their chemical stability and biological activity profiles. Such phytochemical transformations overall serve as the material basis for processing-mediated efficacy regulation, as verified by metabolomic and pharmacodynamic studies [3].

### 3.1. Composition Changes in Citri reticulatae pericarpium (Chenpi)

A study on dried *Chenpi* from seven cultivars (*Citrus reticulata* ‘Succosa’, BDZ; *Citrus reticulata* ‘Kinokuni’, NFMJ; *Citrus reticulata* ‘Dahongpao’, DHP; *Citrus reticulata* ‘Tankan’, JG; *Citrus reticulata* ‘Ponkan’, PG; *Citrus reticulata* ‘Chachi’, GCP; and *Citrus reticulata* ‘Unshiu’, WZMG) reveals similar overall chemical profiles. A total of 53 compounds are identified based on UPLC-MS/MS, among which 48 are flavonoids. Hesperidin and naringin are representative constituents. The total flavonoid content ranges from 36.24 to 88.64 mg/g, and hesperidin accounts for 71~92% of the total content of eight flavonoids [48]. According to the Chinese Pharmacopoeia, hesperidin, nobiletin, and tangeretin are designated as quality markers of dried tangerine peel [5]. Traditional Chinese medicine holds that aging mitigates the “dry-heat property” of dried tangerine peel. Modern research links this property to pro-inflammatory characteristics: aging reshapes its chemical composition, evidenced by decreased hesperidin and increased hesperetin contents. The hesperidin-to-hesperetin ratio may serve as a potential aging marker, which requires further verification. Notably, inter-cultivar differences cannot be ignored for their impacts on the chemical composition of *Chenpi*.

After 1~10 years of aging, flavonoids in *Chenpi* follow a trajectory of initial degradation followed by resynthesis, while polymethoxyflavones (PMFs) continuously accumulate throughout the aging process. Microbial transformation plays a vital role in *Chenpi* aging. The xerophilic genus *Xeromyces* becomes the dominant fungus in the late aging stage and is significantly positively correlated with PMF enrichment, whereas the correlation of *Penicillium* gradually declines. Specifically, *Aspergillus tubingensis* mediates the transformation of nobiletin into 3′-demethylnobiletin, and multiple genera within *Aspergillaceae* jointly participate in the structural modification of flavonoids. These findings provide scientific evidence for microbe-regulated aging processing of *Chenpi* [49]. Core microbes are closely associated with flavor compounds by mediating the accumulation of floral–fruity volatiles and terpenoids, thereby improving *Chenpi* quality and supporting the development of microbe-regulated aging technology [50]. Analysis of 5~30-year-aged *Chenpi* samples using HPLC and ITS sequencing shows that total polyphenols and flavonoids increase with aging, except for hesperidin with a 32.52% decrease, while 5-*O*-desmethylnobiletin increases by 298.36%. *Xeromyces*, *Symmetrospora,* and *Cladosporium* are the dominant fungi. *Xeromyces* accounts for 83.58% and 84.04% in 20- and 30-year-aged samples, respectively, and its abundance is positively correlated with the accumulation of bioactive compounds. As the core genus governing aged tangerine peel quality, it provides insights into the aging mechanism of *Chenpi* [51].

A total of 1092 non-volatile metabolites are identified during *Chenpi* aging, among which 189 exhibit significant variations. These differential metabolites are mainly enriched in phenylpropanoid biosynthesis, flavonoid biosynthesis, and flavone and flavonol biosynthesis pathways. With prolonged aging time, the bitterness, astringency, aftertaste bitterness, aftertaste astringency, and sweetness of *Chenpi* all increase, and the changes in non-volatile metabolite fingerprints dominate flavor formation. Two key findings further clarify the underlying mechanism. First, flavonoid contents increase and then decrease with aging, while the contents of key lignin precursors (sinapyl alcohol and coniferyl alcohol) and most phenolic acids rise, promoting the flavor transition of *Chenpi* from floral–fruity notes to woody notes and enhancing its mellow taste. Second, differential soluble sugars and organic acids are also involved in flavor regulation: sucrose content decreases, whereas other sweet sugars such as stachyose and trehalose increase; key organic acids including D-glucuronic acid and galactaric acid decline, collectively reducing acidity and balancing the overall taste profile [52]. Aging significantly increases the contents of bound phenolics and flavonoids as well as antioxidant activity in *Chenpi*. The elevated contribution of bound phenolics, consisting of 11 characteristic phenolic compounds, is mainly responsible for the superior bioactivity of aged samples. The accumulation of these previously neglected bound phenolics provides a key theoretical basis for developing *Citrus*-peel-derived nutraceuticals and functional foods [53].

Co-fermentation with *Monascus anka* and *Saccharomyces cerevisiae*, a novel processing method for *Chenpi*, mediates the release and transformation of active ingredients via five hydrolases, regulates ten key metabolic pathways, and induces differential metabolism of 55 components. Specifically, free flavonoid contents increase significantly by 48.12%, accompanied by enhanced antioxidant activity. Meanwhile, flavor substances including D-limonene, γ-terpinene, proline, arginine, and serine optimize the sensory quality of *Chenpi*. This technique provides effective technical support for quality improvement in processed *Chenpi* and the development of *Chenpi*-based functional health foods [54].

After stir-frying processing, the essential oil yield of *Chenpi* decreases from 2.11% (raw material) to 1.58% (processed product), and the number of identified chemical components drops from 34 to 32. Meanwhile, the proportion of monoterpene hydrocarbons increases from 83.61% to 93.64%, and the contents of major bioactive compounds, limonene (69.43% → 75.04%) and γ-terpinene (8.93% → 9.94%), both rise significantly. Essential oil from stir-fried *Chenpi* exhibits stronger antioxidant, antibacterial, antitussive, and expectorant activities than that from raw *Chenpi*. These results indicate that traditional stir-frying improves pharmacological activities by regulating the composition and contents of essential oil, endowing processed essential oil with broader application potential in food, pharmaceutical, and chemical industries [55].

Another study demonstrates that the contents of non-volatile bioactive components (synephrine, limonin, phenolic acids, and flavonoids) in *Chenpi* decrease with delayed harvest time, and specific volatile compounds can be used to distinguish samples from different harvesting stages. Phenolic acids, synephrine, and limonin remain stable under various drying temperatures, whereas high-temperature drying at 60 °C significantly triggers compositional transformations of flavonoids (especially polymethoxyflavones) and volatile substances. Most bioactive components decline during the growth of *Chenpi* [56]. Fresh pericarp is recommended to be naturally sun-dried or dried at low temperatures of 30 °C and 45 °C rather than 60 °C to avoid excessive loss of bioactive nutrients. Independent investigation into drying effects reveals that hesperidin content reaches its maximum at 65 °C due to flavonoid metabolic transformation. Temperature and air velocity exert both individual and interactive effects on non-volatile compounds. Considering drying efficiency and product quality comprehensively, the optimal drying condition is 65 °C with an air velocity of 6 m/s [57]. A dual-wavelength HPLC method is established for simultaneous quantitative analysis of hesperidin and five polymethoxylated flavones in *Chenpi*. Dynamic monitoring over a three-year storage period at 25 °C shows that hesperidin content decreases continuously, while all polymethoxylated flavone components and antioxidant capacity increase progressively. These findings verify that polymethoxylated flavones can serve as indicators for quality variation in *Chenpi* during storage, and long-term storage helps improve *Chenpi* quality [58].

### 3.2. Composition Changes in Citri grandis exocarpium (Huajuhong)

As a traditional medicinal and edible herb, the pharmacological efficacy of *Huajuhong* is closely associated with its active ingredient composition, aging duration, and processing technologies. A series of studies concerning drying-mediated quality regulation, non-destructive identification, and metabolomic aging mechanism have been summarized herein. These studies have systematically illustrated the quality formation rules of *Huajuhong* and provide a theoretical basis and technical support for its efficient industrial processing, precise quality control, and value improvement.

Drying serves as a core processing procedure of *Huajuhong*. Drying methods, temperatures, microwave intensities, and humidity control modes directly determine the retention rate of bioactive constituents and the final product quality. Microwave–vacuum drying with microwave intensities ranging from 0.75 to 2.00 W/g would significantly affect the contents of flavonoids, including naringin and rhoifolin. Flavonoid contents would decline with increased microwave intensity at low levels, while total flavonoids, naringin, and rhoifolin are elevated at 2.00 W/g due to the formation of small-molecular phenolic compounds induced by the drying temperature. Combined with drying kinetics and microstructure analyses, microwave–vacuum drying could regulate the drying characteristics and overall quality of *Huajuhong*, supporting its efficient and high-quality industrial production [59]. Compared with hot air drying under constant relative humidity control (HAD-CRHC), hot air drying with alternating relative humidity control (HAD-ARHC) would markedly increase flavonoid accumulation in *Huajuhong*. Under optimized step-wise HAD-ARHC conditions, the flavonoid content would reach 121.94 mg/g, realizing bioactive component enrichment along with improved drying efficiency and product quality [60]. Dynamic changes in *Huajuhong’s* physicochemical properties under different drying temperatures indicate that structural damage occurs and flavonoids, polysaccharides, and volatile oils degrade when the moisture content decreases from 1.0000 to 0.2175 g/g (dry basis), whereas naringin and rhoifolin contents increase. Higher drying temperatures would accelerate the above variations. After the moisture content drops below 0.2175 g/g, volatile oil species increase with newly generated terpenes, alcohols, aldehydes, and esters. Overall, total active ingredients would decrease before the moisture reaches 0.2175 g/g, followed by elevated metabolite contents and species during subsequent drying [61]. Notably, the white frost (WF) on *Huajuhong*, mainly consisting of crystalline naringin (approximately 80% of the total mass), originates from endogenous flavonoid crystallization rather than microbial contamination. Drying-induced shrinkage and rupture of oil glands trigger metabolite migration and surface crystallization for WF formation. Naringin can be efficiently extracted by 30 min of water boiling, with higher yields in WF samples than non-frost (NF) samples. WF extracts exhibit superior anti-inflammatory activity by more potently suppressing lipopolysaccharide-induced NO production and the secretion/transcription of TNF-α, IL-6, IL-1β, iNOS, and NF-κB, suggesting that flavonoid crystallization enhances the anti-inflammatory capacity of *Huajuhong* [62]. Notably, visible white frost can only be observed on the surface of just over 30% of commercial samples, indicating that white frost does not form on all *Huajuhong* specimens and cannot be regarded as a universal characteristic.

The pharmacological activities of *Huajuhong* are highly dependent on chemical composition variations during aging, yet conventional analytical methods fail to accurately identify aging years for quality control. A feature-optimized classification framework integrating hyperspectral imaging (HSI) and a Broad Learning System (BLS) has therefore been established for rapid non-destructive aging discrimination. This dual-side spectral validation strategy provides a rapid, non-destructive, and reliable approach for aging-year authentication of *Huajuhong*, facilitating the modernized quality supervision of medicinal–edible herbs [63].

*Huajuhong* possesses prominent antioxidant and anti-inflammatory properties, whereas chemical compositional variations and antioxidant activity alterations during storage-induced aging remain poorly understood. Comprehensive metabolomics has been applied to investigate metabolic changes in CGE samples aged for 1 (CG1), 3 (CG3), and 5 years (CG5). A total of 1249 metabolites were detected, while 57 differential metabolites, including naringin, were screened as potential aging markers via chemometric analyses. KEGG annotation, enrichment, and topological analyses reveal flavonoid biosynthesis as the pivotal metabolic pathway during CGE aging, with 14 differential flavonoids identified. Total flavonoids, naringin, naringenin, butin, 7′,4-dihydroxyflavone, and phlorizin have been validated as key metabolites correlated with antioxidant activity. These findings elucidate how aging time regulates flavonoid accumulation and antioxidant capacity, providing scientific evidence for the aging mechanism of CGE [64].

### 3.3. Composition Changes in Aurantii fructus immaturus (Zhishi) and Aurantii fructus (Zhiqiao)

Recent studies have investigated the chemical profiling variations in *Aurantii fructus immaturus* (*Zhishi*) before and after bran-frying. Chromatographic–mass spectrometric approaches combined with chemometrics are widely adopted to characterize processing-induced compositional changes. HPLC fingerprinting is employed and integrated with chemometric analysis. In raw *Zhishi*, 26 common peaks are detected*,* with similarity coefficients ranging from 0.927 to 0.996. In bran-fried samples, 33 common peaks are found, with similarities of 0.969~0.997. Additionally, nine characteristic differential markers are further screened out. Quantitative determination indicates significant reductions (*p* < 0.05) in the contents of rhoifolin (from 1.37% to 0.79%), narirutin (from 1.44% to 1.22%), naringin (from 5.12% to 4.58%), hesperidin (from 0.76% to 0.66%) and neohesperidin (from 1.53% to 1.18%). In contrast, the levels of nobiletin (from 2.34% to 2.65%) and tangeretin (from 1.74% to 2.33%) are markedly elevated following bran-frying [65]. Using UPLC-Q-TOF-MS coupled with PCA, OPLS-DA and VIP analyses, raw, bran-fried and scalded *Zhishi* are comparatively evaluated. A total of 42 chemical components are preliminarily identified, among which 22 exhibit significant content discrepancies. Specifically, 11, 15 and 11 differential components are distinguished between raw and bran-fried samples, raw and scalded samples, as well as bran-fried and scalded samples, respectively [66]. The majority of constituents are increased after bran-frying, except syringetin-3-*O*-glucoside, which decreases due to high-temperature-mediated glycosidic bond cleavage, along with the upregulation of 5,7,8,4′-tetramethoxyflavone. Similarly, most compounds are enriched after scalding, while feruloylputrescine declines as a result of thermal oxidation. Five common differential components including adenosine are upregulated post-processing, which is hypothesized to be associated with the pungent–bitter medicinal nature of *Zhishi*. Six *Citrus*-specific polymethoxyflavonoids are identified as discriminative markers between bran-fried and scalded products [32]. UPLC-Q-TOF-MSᴱ combined with the UNIFI database and multivariate statistical analysis identifies 64, 58 and 18 chemical constituents in raw *Zhishi*, bran-fried *Zhishi* and processed wheat bran excipient, respectively, and screens 19 processing-related differential markers mainly assigned to volatile oils, flavonoids, phenolic acids, coumarins and saponins [67]. Collectively, these investigations demonstrate that bran-frying can mitigate the drastic medicinal property of *Zhishi* by regulating the composition and abundance of characteristic chemical constituents, particularly flavonoids. Such analytical strategies provide robust technical support and experimental evidence for quality evaluation, material basis elucidation and processing mechanism exploration of bran-fried *Zhishi*.

Modern analytical techniques including internal extractive electrospray ionization mass spectrometry (iEESI-MS), HPLC, UPLC-Q/TOF-MS and UHPLC-high-resolution tandem mass spectrometry are applied to systematically investigate chemical changes in *Aurantii fructus* (*Zhiqiao*) induced by bran-stir-frying, salt-processing, vinegar-processing, fermentation, steaming and Jiangxi Zhangbang-style characteristic processing. Combined with multivariate statistical analysis, iEESI-MS confirms that flavonoids are the dominant differential metabolites, accounting for two-thirds of total altered compounds, which can serve as vital markers for origin discrimination and quality evaluation [68]. HPLC quantification reveals that the total contents of flavonoids and coumarins increase after processing. The dominant flavonoids follow the order: water-moistened sample > bran-stir-fried sample > salt-processed sample > vinegar-processed sample > raw sample [69]. HPLC fingerprinting further verifies distinct regulatory effects of different processing methods on flavonoid profiles [70]. Using the AHP-entropy weight method, the optimal fermentation parameters for Lingnan-style *Zhiqiao* are determined as 4 h soaking, 48 h fermentation at 37 °C and 90% humidity. After fermentation, flavonoid glycosides decrease and aglycones are significantly elevated [71]. Fourteen flavonoid-based differential markers are screened via UPLC-Q/TOF-MS, and Zhangbang-style bran-stir-fried and honey-processed *Zhiqiao* is verified as the optimal processed product with the highest active ingredient contents [72]. Bran-stir-frying reduces d-limonene and increases 5-hydroxymethylfurfural, improves microstructure, and enhances antioxidant, anti-inflammatory and gastrointestinal motility-promoting activities [73]. Fermented-steaming modulates flavor characteristics, enriches beneficial intestinal microbes and inhibits pathogenic bacteria of *Zhiqiao* [74]. Auraptene, the key differential component between raw and bran-stir-fried samples, exerts bidirectional regulation on gastrointestinal smooth muscle by inhibiting acetylcholinesterase, thereby mediating the medicinal property transformation from soothing liver-*qi* stagnation to harmonizing stomach and improving digestion [75]. UHPLC–high-resolution tandem mass spectrometry demonstrates that fermentation and high temperature promote deglycosylation of flavonoid glycosides, and the optimal fermented-steaming condition is 3-day fermentation combined with 3~4 h steaming [76]. Taken together, these studies provide systematic evidence for a quality evaluation and mechanism elucidation of processed *Zhiqiao* from the aspects of chemical dynamics, process optimization, material basis and functional mechanisms.

### 3.4. Composition Changes in Citri reticulatae semen (Juhe)

Several studies systematically characterize the component conversion, color variation, and anti-inflammatory activity of *Juhe* during processing. HPLC combined with color difference analysis reveals that during salt-processing and stir-frying, limonin remains stable. Nomilin decreases significantly, while obacunone increases markedly, and their contents are correlated with chromatic parameters. Nomilin is thermally converted to obacunone at 200~250 °C, and obacunone exerts stronger anti-inflammatory activity against LPS-induced NO release in RAW264.7 cells than nomilin [40]. Another HPLC study finds that processing reduces the contents of limonin and nomilin. The salt-processed and stir-fried products show greater losses than the bran-fried ones. The salt-processed *Juhe* presents unstable batch-to-batch quality, despite the enhanced analgesic and anti-inflammatory effects [41]. UPLC-Q-TOF/MS analysis indicates that the levels of eriocitrin, limonin, nomilin, and obacunone are elevated in salt-processed *Juhe*. Limonin, nomilin, and obacunone are potential markers for distinguishing raw and processed samples [19]. In another study, 16 chemical constituents (limonoids, flavonoids, phenolic acids, coumarins, and terpenoids) are systematically isolated and identified from the 85% ethanol extract of salt-processed *Juhe*. Four compounds are first isolated from the *Citrus* genus, and three are newly detected in *Juhe* specifically. Antioxidant tests show that epicatechin possesses the strongest radical-scavenging activity, and salt-processing notably improves the total antioxidant capacity of the extract, laying a material foundation for investigating its synergistic mechanism [77]. This finding expands the chemical database of salt-processed *Juhe* and highlights the processing-driven enhancement of bioactivity.

Using five characteristic flavonoids as quality markers, a study confirms that their contents are significantly affected by the storage and aging time of *Juhe*. Storage temperature and humidity reshape its volatile organic compound profiles. Eighteen aging-period markers and eight spoilage indicators are identified. Hesperidin maintains a stable bioaccessibility of 30~50% regardless of aging time and gender, offering key evidence for *Juhe* quality control and digestion-related research [78]. It provides a practical reference for standardizing the post-harvest preservation and quality evaluation of *Juhe*.

In general, during processing procedures including concocting, aging, drying and fermentation, all *Citrus* MEHHs exhibit predominant chemical variations in flavonoids, which commonly undergo glycosidic hydrolysis, oxidation and polymerization alongside continuous accumulation of polymethoxyflavones, leading to improved overall bioactivities. Distinct characteristic marker compounds are observed among different species: hesperidin dominates in *Citri reticulatae pericarpium*, naringin serves as the key constituent in *Citri grandis exocarpium*, *Aurantii fructus immaturus* and *Aurantii fructus* are rich in mixed flavonoids and coumarins, while limonoids are the signature components of *Citri reticulatae semen*. Processing induces divergent transformation pathways: aging of dried tangerine peel relies on microbe-mediated metabolism, exocarpium of pummelo peel is prone to crystallization of active ingredients, bran-stir-frying and other thermal processing moderate the medicinal properties of immature and mature *Citrus* fruits, and thermal isomerization of limonoids primarily occurs in *Citrus* seeds. Temperature, moisture content and microorganisms act as three pivotal factors regulating chemical profiles, sensory properties and pharmacological effects, laying a fundamental basis for processing optimization and quality control of *Citrus* MEHHs (Table 3).

## 4. Processing Mechanisms of *Citrus* MEHHs

Processing of *Citrus* medicinal and edible homologous herbs modulates their therapeutic efficacy through chemical composition alterations, with underlying mechanisms encompassing chemical transformation and pharmacodynamic regulation. This section integrates traditional Chinese medicine (TCM) theory with contemporary scientific research to systematically elucidate the processing mechanisms of *Citrus* herbs from phytochemical and pharmacological perspectives. Chemical transformation of *Citrus* herbs during processing constitutes the material basis for efficacy regulation, primarily involving hydrolysis, oxidation, polymerization, and isomerization reactions. These reactions are modulated by processing parameters (temperature, duration, humidity) and auxiliary materials, inducing changes in active component content, structural conformation, and aqueous solubility.

### 4.1. Hydrolysis

Hydrolysis is the core chemical reaction dominating the compositional transformation and bioactivity improvement of *Citrus* medicinal materials during processing and preparation. Multiple processing methods, including enzymatic hydrolysis treatment and thermal roasting processing, can trigger hydrolysis reactions of carbohydrate, flavonoid glycoside and polysaccharide substances in *Citrus* materials, thereby breaking original chemical structures, reconstructing material compositions, and ultimately optimizing the functional and pharmacological activities of *Citrus* products.

Enzymatic hydrolysis serves as a critical biological processing approach for *Citrus* materials, firstly disrupting plant cell wall structures to depolymerize and transform bound active ingredients, inducing comprehensive reconstruction of internal chemical compositions. Relevant studies have verified that *Citrus* samples treated by enzymatic hydrolysis exhibit significantly superior active substance content and biological activity compared with control groups such as essential oil residues and ultrasound-assisted extraction samples. Specifically, the total phenolic content and superoxide dismutase (SOD)-like activity of hydrolyzed and oven-dried *Citrus* samples reach 3688 mg GAE/100 g and 3505 U/g respectively, which are markedly higher than those of essential oil residues. Meanwhile, the hydrolyzed samples achieve a maximum inhibitory rate of 93.77% against carbohydrate hydrolyzing enzymes, outperforming the ultrasound-assisted extraction group. In terms of characteristic phenolic components, the relative contents of sinapic acid and esculetin in hydrolyzed *Citrus* materials reach 47.33–59.59% and 16.85–18.31% respectively [79]. The structural modification of active substances induced by enzymatic hydrolysis effectively enhances the hypoglycemic and anti-obesity bioactivities of *Citrus* materials.

For immature *Citrus* peel, enzymatic processing specifically triggers the targeted hydrolysis of flavonoid glycosides. The cleavage of glycosidic bonds during the reaction converts bound flavonoid glycosides into free flavonoid monomers (mainly hesperetin and naringenin), and simultaneously promotes the accumulation of phenolic active substances. The progress of this glycoside hydrolysis reaction is significantly regulated by processing parameters: enzyme dosage, reaction temperature and duration positively drive the chemical conversion of flavonoid glycosides and effectively increase the contents of hesperetin, naringenin and total phenolics. However, excessive enzymatic reaction will destroy the structural integrity of active components, thereby reducing the DPPH radical scavenging activity of the product. Under the optimal processing conditions (4% enzyme concentration, 51 °C, 18 h), sufficient and thorough hydrolysis of flavonoid glycosides can be realized. After enzymatic hydrolysis, the contents of hesperetin, naringenin and total phenolics in immature *Citrus* peel are increased by 251.7 times, 45.5 times and 2.6 times respectively [80]. The significant chemical transformation driven by enzymatic hydrolysis endows *Citrus* hydrolysate with excellent development potential as high-value functional food.

In addition to fresh *Citrus* materials, *Citrus* processing waste, which accounts for 45–55% of the total fruit weight and is rich in fermentable sugars, provides a favorable material basis for hydrolysis transformation and resource recycling. The hydrolysis-based processing can directionally decompose waste biomass through the cleavage and recombination of glycosidic bonds and polysaccharide chains, realizing the reconstruction of internal component structures of *Citrus* waste. This chemical transformation can produce a variety of high-value active substances, including organic acids, pectin, and enzymes, as well as bioethanol and bio-oil [81]. Compared with chemically synthetic products, the natural active ingredients derived from hydrolysis of *Citrus* waste have better physicochemical properties and broader application advantages in food and pharmaceutical industries. Moreover, these natural hydrolyzed products can replace corrosive industrial chemicals, not only reducing environmental pollution but also significantly improving the utilization value of *Citrus* waste raw materials.

Apart from enzymatic hydrolysis, thermal processing represented by roasting can also induce hydrolysis reactions in *Citrus* medicinal materials, taking *Chenpi* as a typical example. During the roasting process, high temperature triggers the hydrolysis of carbohydrates in *Chenpi*, leading to a reduction in total sugar content. The thermal hydrolysis reaction further promotes the enrichment of phenolic and flavonoid active components; when the roasting temperature is 155 °C, the contents of narirutin and hesperidin in the product reach 15.32 mg/g and 11.50 mg/g respectively. In addition, roasting-induced hydrolysis acts synergistically with the Maillard reaction, which greatly increases the yields of DDMP and 5-hydroxymethylfurfural. The joint reconstruction of material compositions by hydrolysis and thermal chemical reactions optimizes the substance profile of *Chenpi* extract and improves its antioxidant capacity, among which DDMP exhibits the strongest DPPH radical scavenging activity [82].

### 4.2. Oxidation

Oxidation is a core chemical reaction throughout *Citrus* herb and by-product processing, which alters the structural integrity and chemical composition of endogenous flavonoids, phenolic acids, and essential oils, fundamentally determining the quality and functional stability of *Citrus*-derived products. This section focuses on the oxidation mechanisms induced by different processing and modification treatments in *Citrus* systems.

The oxidative degradation of lemon oil active components is governed by synergistic catalytic and environmental oxidation mechanisms. Trace copper ions act as a chemical catalyst to initiate intensive oxidation of monoterpene components including α-pinene, β-pinene and γ-terpinene under aerobic conditions, while antioxidants (BHA, tocopherol) can block this catalytic oxidation pathway. Photo-thermal coupled oxidation exhibits component-dependent reaction regularity: γ-terpinene and limonene follow consistent oxidative degradation pathways under UV-aerobic conditions and copper-catalyzed thermal oxidation, generating identical degradation products. In contrast, citral undergoes exclusive photo-specific oxidation to form photocitrals, with a reaction mechanism distinctly different from thermal oxidation [83]. This demonstrates that temperature and oxygen jointly regulate the selective oxidative transformation of volatile components during lemon oil processing.

Thermal induction and long-term storage trigger oxidative non-enzymatic browning in *Citrus* juice, a quality-deteriorative oxidation reaction driven by precursor polymerization and intermediate accumulation. The oxidation of endogenous amino acids and sugars produces 2,5-dimethyl-4-hydroxy-3(2*H*)-furanone, a characteristic oxidative marker that directly reflects the browning degree of *Citrus* juice. Two targeted inhibition mechanisms suppress this oxidative browning reaction: ion exchange removes amino acid and protein precursors to terminate the initial oxidation reaction; sulfiting agents inhibit the oxidative generation of furfural and 5-hydroxymethylfurfural intermediates, thereby blocking the subsequent browning cascade reaction and stabilizing the oxidative balance of *Citrus* juice components [84].

Controlled selective oxidation enables directional structural modification of *Citrus* waste cellulose, with clear radical and grafting reaction mechanisms. A mild room-temperature aqueous system utilizes TEMPO catalysis coupled with hypochlorite–periodate composite oxidation to trigger selective oxidation of cellulose molecular chains, achieving directional grafting of carboxyl groups on native cellulose skeletons. The mild oxidative condition only modifies the cellulose chemical structure without inducing oxidative loss of saturated fatty acids in *Citrus* waste. The retained hydrophobic fatty components and oxidized cross-linked nanostructure jointly change the material’s interfacial properties: oxidative structural rearrangement forms disordered entangled molecular chains, while hydrophobic substances endow the modified cellulose with water resistance and network-forming capacity, realizing functional upgrading via targeted oxidative modification [85].

Electric field physical processing regulates *Citrus* component oxidation via enzyme inactivation and structural physical intervention mechanisms. Gradient voltage in electrohydrodynamic drying destroys *Citrus* peel tissue and cell membrane structures, directly inducing voltage-dependent oxidative degradation of endogenous phenolics, carotenoids and volatile compounds. Compared with direct current drying, alternating current drying optimizes the oxidation regulation mechanism: it shortens processing duration to reduce thermal oxidative accumulation, and effectively inactivates oxidative enzymes in *Citrus* peel, thereby inhibiting enzyme-mediated endogenous oxidative deterioration and maintaining the structural and compositional stability of natural bioactive components [86].

Pyrolysis temperature modulates the interfacial oxidation mechanism of *Citrus* peel biochar, realizing synergistic adsorption–electrochemical oxidative degradation of pollutants. High-temperature pyrolysis (800 °C) constructs abundant oxygen vacancy active sites and persistent free radical structures on biochar surface, which are the core functional domains for electrochemical oxidation. Under an applied electric field, these active sites activate dissolved oxygen to generate massive superoxide radicals, triggering efficient oxidative reactions. The oxidative degradation mechanism of dimethyl phthalate follows a stepwise structural transformation pathway, including demethylation, dehydroxylation, hydroxylation and decarboxylation. The optimized interfacial structure of high-temperature biochar eliminates mass transfer limitations, accelerates the coupled adsorption–oxidation reaction kinetics, and achieves efficient mineralization of organic micropollutants [87].

Thermally modified *Citrus* peel supports selective catalytic oxidation via an interfacial redox regulation mechanism. The porous structure of thermally treated *Citrus* peel optimizes the dispersion and interfacial microenvironment of immobilized Keggin-type phosphomolybdenum heteropolyacid, eliminating the solubility limitation of polar catalytic systems. With hydrogen peroxide as a green oxidant, the heteropolyacid-based catalytic system drives reversible redox electron transfer, enabling precise selective oxidation of sulfides to sulfoxides. This interfacial oxidation regulation mechanism effectively restrains over-oxidation side reactions, achieving high-selectivity oxidative conversion of target substrates [88].

### 4.3. Polymerization and Isomerization

During the processing and thermal treatment of medicinal and edible *Citrus* resources, endogenous active components including terpenoids, flavonoids, and phenolic acids are subjected to thermal effects, physical actions, and medium regulation. These components commonly undergo isomerization, polymerization, and coupled degradation reactions, resulting in molecular structural remodeling and component content fluctuation. Such chemical changes serve as the core internal mechanism determining the pharmacological activity and quality characteristics of processed *Citrus* products [89,90]. Existing studies have clarified the component-specific rules of these two key reactions in the transformation of critical *Citrus* constituents, providing an essential theoretical basis for explaining the changes in processing properties and pharmacological efficacy of *Citrus* materials.

Limonene, a representative terpenoid in *Citrus*, undergoes isomerization as the dominant transformation pathway under catalytic processing conditions. Reaction duration critically balances the generation of isomeric products and polymeric by-products [91]. It has been confirmed that the Ti-MCM-41 catalyst promotes the skeletal isomerization of R(+)-limonene, primarily producing terpinolene, along with α-terpinene and γ-terpinene as secondary products. The three terpinene intermediates can be further converted into *p*-cymene via dehydrogenative aromatization. Short-duration processing favors the isomerization pathway and selectively accumulates limonene isomers. In contrast, prolonged processing facilitates deep dehydrogenation but simultaneously induces oxidative and polymeric side reactions, generating polymeric impurities and reducing product purity. This study clearly illustrates the time-dependent transformation characteristics of *Citrus* terpenoids, wherein isomerization dominates under mild conditions and polymerization acts as a secondary adverse reaction under excessive processing.

Green catalytic processing further verifies the efficient isomerization performance of *Citrus*-derived limonene [92]. In a solvent-free green system employing glucose-based sulfonated carbon as a metal-free biocatalyst, the textual properties and surface acidic groups of the catalyst effectively drive limonene isomerization. Under processing conditions of 150 °C for 2 h, the limonene conversion rate reaches 96%, and the total yield of major terpenoid isomers ranges from 40% to 50%. This study demonstrates that mild and green processing conditions enable the directional isomerization of *Citrus* terpenoids while avoiding disordered polymerization and degradation induced by harsh reactions, supporting the quality control of *Citrus* terpenoids during green processing.

Beyond independent isomerization, *Citrus*-derived limonene achieves coupled isomerization and polymerization during multi-step modified processing, realizing high-value structural transformation from monomers to macromolecular polymers [93]. Specifically, limonene undergoes catalytic activation and saponification-induced isomerization to form aldehyde intermediates, which are further converted into acrylate monomers via multi-component reactions. Finally, free radical polymerization initiated by AIBN produces high-molecular-weight polyacrylates. This finding reveals that targeted process regulation can integrate isomeric structural modification and controllable polymerization of *Citrus* terpenoids, updating the conventional understanding that terpenoids only undergo simple isomerization during processing.

Unlike terpenoid reaction characteristics, polymerization serves as the predominant modification pathway for *Citrus* flavonoids and phenolic acids during thermal processing, accompanied by synergistic isomerization and degradation reactions that cause active component loss and efficacy attenuation [94]. Thermal processing (90 °C for 10 min) of *Citrus grandis* juice triggers the hydrolysis of flavonoid glycosides, as well as functional group isomerization, molecular rearrangement, and polymerization of endogenous flavonoids and phenolic acids. These reactions lead to significant losses of total polyphenols, total flavonoids, and carotenoids, with maximum loss rates of 40%, 45%, and 25%, respectively. Structural isomerization and polymerization degradation of characteristic flavonoids (e.g., naringin) are the key causes of decreased antioxidant, α-amylase inhibitory, α-glucosidase inhibitory, and pancreatic lipase inhibitory activities of processed *Citrus* products. Additionally, such component transformation exhibits cultivar specificity, which guides the quality regulation of *Citrus* processing.

**Figure 2:**
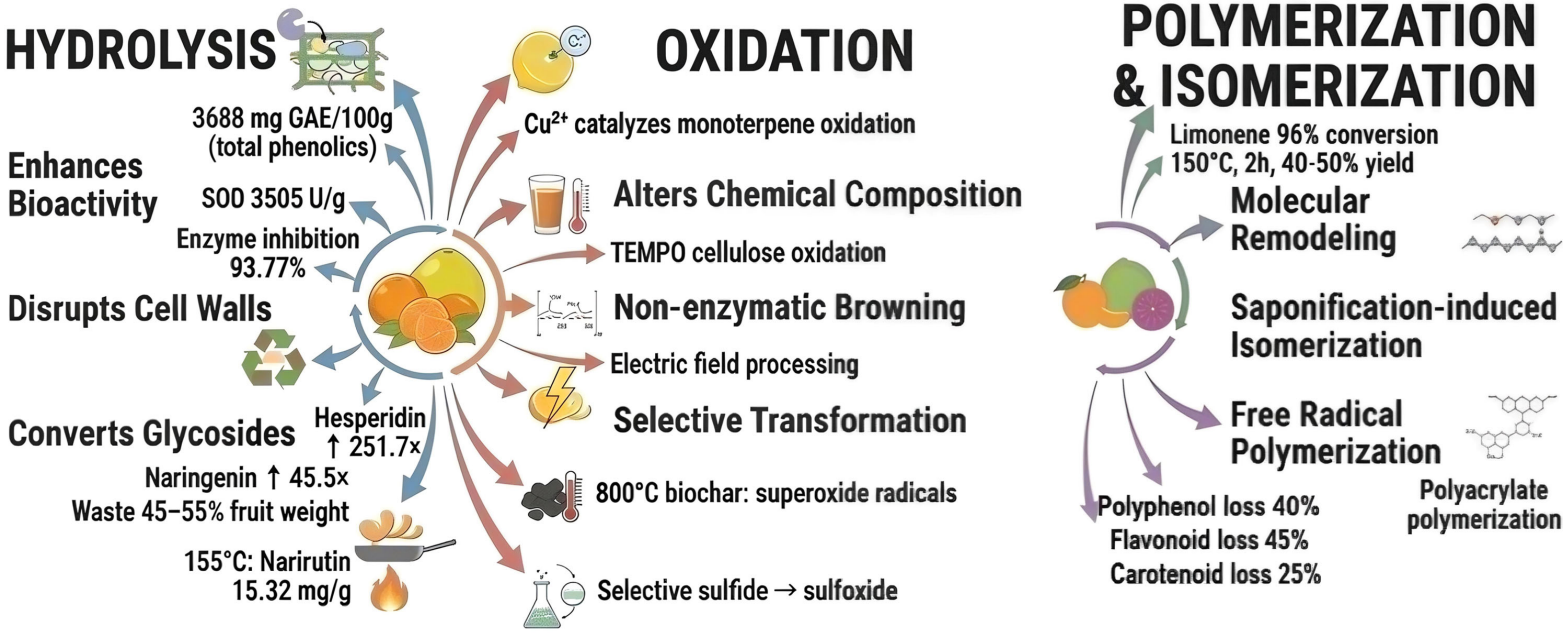
Main chemical transformation mechanisms during *Citrus* processing.

Taken together, terpenoids preferentially undergo directional isomerization, while excessive processing triggers undesirable polymeric side reactions. Flavonoids and phenolic acids are thermosensitive, with thermal-induced polymerization, isomerization, and degradation as their major modification pathways. The component-specific and process-dependent characteristics of isomerization and polymerization fundamentally determine the changes in medicinal properties, biological activity, and product quality during *Citrus* processing (Figure 2).

## 5. Conclusions

The present review summarizes key research advances in processing technologies, processing-induced phytochemical changes, and functional mechanisms of five typical *Citrus* medicinal and edible homologous herbs (MEHHs): *Citri reticulatae Pericarpium* (*Chenpi*), *Citri grandis Exocarpium* (*Huajuhong*), *Aurantii Fructus Immaturus* (*Zhishi*), *Aurantii Fructus* (*Zhiqiao*), and *Citri Reticulatae Semen* (*Juhe*). As valuable TCM-derived resources with dual food and medicinal properties, *Citrus* MEHHs contain abundant bioactive compounds, mainly flavonoids, limonoids, essential oils, and phenolic acids. These components confer antioxidant, anti-inflammatory, gastrointestinal regulatory, and other pharmacological activities, underpinning their long-term use in traditional healthcare and modern functional food and pharmaceutical industries [1,2].

Processing is a core procedure governing the quality and efficacy of *Citrus* MEHHs, including traditional empirical and modern innovative processing systems. Traditional methods such as natural aging, sun-drying, stir-frying, salt-processing, and microbial fermentation are tailored to the inherent botanical and chemical traits of *Citrus* herbs [43]. Through long-term natural biotransformation and empirical optimization, these conventional approaches improve efficacy, reduce toxicity, and optimize sensory properties, embodying the holistic philosophy of TCM processing. However, these empirical methods are constrained by environmental conditions and manual operation differences, featuring long production cycles, poor repeatability, and difficulty in precise quality control. Meanwhile, the random loss of thermosensitive active components also restricts the stable play of herbal efficacy.

In contrast, modern technologies (low-temperature, vacuum, microwave, freeze, and composite hybrid drying) overcome the limitations of traditional processing, including long cycles, poor batch consistency, and loss of thermolabile active ingredients. These advanced methods boost processing efficiency, stabilize product quality, and enable directional retention and enrichment of bioactive compounds, supporting standardized, large-scale, and high-quality industrial production of *Citrus* MEHH products [95]. Precise regulation of processing temperature, time, and environment realizes directional retention and enrichment of bioactive components, unifies product quality, and greatly improves production efficiency and batch stability, providing feasible technical support for the modern industrialization of *Citrus* MEHHs [96].

Processing-induced phytochemical transformation constitutes the fundamental material mechanism for the efficacy regulation of *Citrus* MEHHs. Key parameters (temperature, duration, humidity, processing adjuvants) trigger hydrolysis, oxidation, polymerization, and isomerization, remodeling the composition, structure, and solubility of endogenous bioactive components. Major variations include the hydrolysis of flavonoid glycosides into high-bioavailability aglycones, accumulation and structural modification of polymethoxyflavones during aging, conversion of low-activity limonoids (e.g., nomilin) to high-potency obacunone, and optimization of volatile oil and flavor profiles. Such compositional changes alter the medicinal, sensory, and biological properties of *Citrus* herbs, scientifically interpreting the classic TCM principle that “processing modulates herbal efficacy”.

## 6. Limitations and Future Perspectives

Previous research has investigated both conventional and modern processing methods for *Citrus* medicinal and edible homologous herbs. A range of pharmacological properties including antioxidant, anti-inflammatory and gastrointestinal protective effects have been validated through in vitro and in vivo assays, and major bioactive constituents have been partially characterized. Even so, industrial translation of these herbal materials is held back by four key limitations. No consistent processing protocols or universal quality benchmarks are available at present. Variable production parameters from different regions and manufacturers bring inconsistent phytochemical compositions and unstable therapeutic effects, slowing down standardized industrial production. The multi-layer regulatory pathways triggered by processing are not fully understood, covering interactions among diverse metabolites, microbe-mediated biotransformation, and the causal link between chemical shifts and bioactivity, which prevents rational, efficacy-guided refinement of processing techniques. Most pharmacological observations rely on preclinical data, while well-controlled human trials are scarce to confirm clinical efficacy and long-term safety. Furthermore, flavonoid glycosides show poor oral absorption, restricting their further development into high-value functional food ingredients and pharmaceutical formulations.

Future research into *Citrus* medicinal and edible homologous herbs will center on systematic construction of unified quantitative processing specifications and universal quality control benchmarks to mitigate cross-regional and cross-manufacturer discrepancies in technical parameters and end-product consistency. Multi-omics profiling coupled with microbial community analysis will be deployed to untangle complex phytochemical interconversion networks and microorganism-driven biotransformation cascades, further elucidating the causal correlation between processing-induced compound remodeling and corresponding pharmacological performance so as to underpin targeted precision processing protocols. Sustained efforts shall be dedicated to translational investigation via well-designed clinical trials for rigorous validation of the efficacy and safety of processed *Citrus* medicinal materials, alongside development of advanced delivery formulations to ameliorate the limited oral bioavailability of dominant flavonoid glycosides and unlock high-value applications in functional food supplements and therapeutic preparations. Integrated, intelligent, eco-friendly processing frameworks combining mild composite drying, controlled microbial fermentation and standardized auxiliary stir-frying technologies will also be refined to achieve directional retention of characteristic bioactive constituents, shortened production cycles and reproducible automated manufacturing, thereby inheriting the empirical core of classical TCM Paozhi while advancing modern industrial production.

## Figures and Tables

**Table 1:** Comparison of traditional processing methods for major *Citrus* MEHHs.

Species	Chinese Name	Source	TCM Nature	TCM Therapeutic Effects	Pharmacological Activity	Main Processed Products in TCM
*Citri reticulatae pericarpium*	*Chenpi* (陈皮)	Dried pericarp of *C. reticulata* Blanco and its cultivars	Warm; pungent, bitter; spleen, lung meridians	Regulates *qi*, invigorates spleen, eliminates dampness, resolves phlegm	Regulates gastrointestinal function, anti-inflammation, expectorant, antioxidant	Bran-fried: Mild dryness, enhanced spleen–stomach harmonizing; Charred: Regulates *qi*, invigorates spleen, astringes to stop bleeding.
*Citri grandis exocarpium*	*Huajuhong* (化橘红)	Dried outer peel of *C. grandis* var. *tomentosa* or *C. grandis*	Warm; pungent, bitter; lung, stomach meridians	Antitussive, expectorant, regulates *qi*, relieves distension	Antitussive, expectorant, anti-inflammatory, airway-protective	Bran-fried: Reduces dryness, mild phlegm-resolving and *qi*-regulating properties; Honey-fried: Moistens lung, resolves phlegm, relieves cough.
*Aurantii fructus immaturus*	*Zhishi* (枳实)	Dried young fruits (2–3 cm, May–June)	Warm; pungent, bitter, sour; spleen, stomach meridians	Breaks *qi*, resolves stagnation, reduces distension, softens masses	Promotes gastrointestinal peristalsis, anti-inflammatory, antioxidant	Bran-fried: Mild *qi*-breaking, enhanced food-eliminating and spleen-invigorating; Charred: Astringes to stop bleeding, regulates *qi*, eliminates stagnation.
*Aurantii fructus*	*Zhiqiao* (枳壳)	Unripe fruits (July–August)	Warm; pungent, bitter, sour; spleen, stomach, large intestine meridians	Regulates *qi*, relieves distension, resolves stagnation	Moderate gastrointestinal regulation, anti-inflammatory, antioxidant	Bran-fried: Mild nature, enhanced spleen invigoration and distension relief; Charred: Regulates *qi* and stops bleeding.
*Citri reticulatae semen*	*Juhe* (橘核)	Dried seeds of *C. reticulata* and its cultivars	Warm; pungent, bitter; liver, kidney meridians	Regulates *qi*, relieves pain, resolves masses, soothes liver	Analgesic, anti-inflammatory, mass-resolving, liver-soothing, antioxidant	Salt-fried: Guides to kidney, enhances mass-dissipating and pain-relieving; Stir-fried: Mild nature, regulates *qi* and relieves pain.

*The pharmacological activities are mainly derived from preclinical studies such as animal and cell experiments, and relevant clinical evidence remains insufficient at present.*

**Table 2:** Comparison of TCM and modern processing methods for different *Citrus* MEHHs.

MEHH	Processing Type	Core Procedures	Key Parameters	Mechanisms	Changes in Processing	Advantages & Limitations	References
*Chenpi*	Traditional	Harvesting → peeling → drying → aging; carbonization, earth-frying, bran-frying, salt-processing	Harvesting: October–December; sun-dried moisture: 12–15%; aging ≥3 years; stir-frying temperature: 100–220 °C; 2% NaCl solution for salt-processing	Natural fermentation and microbial transformation; thermal modification, ionic regulation, mineral adsorption	Phenolics and flavonoids enriched by aging; carbonization enhances hemostatic and antidiarrheal effects; bran-frying alleviates irritation; salt-processing improves meridian tropism and analgesia	Well-established empirical basis and mild medicinal properties; long aging cycle, unstable batch quality, vulnerable to environmental interference	[5,13,14,15,16,17,18,19]
	Modern	Low-temperature drying, vacuum drying, infrared drying; controlled temperature–humidity aging, microbial fermentation aging	Low-temperature drying: 40–60 °C; controlled aging: 20–25 °C, humidity 60–70%	Mild drying reduces degradation of bioactive components; artificial regulation accelerates component transformation	Retention of volatile oils and flavonoids; shortened aging period and improved batch consistency	High efficiency and controllable quality; high equipment cost	[20,21,22,23,24]
*Huajuhong*	Traditional	Harvesting → peeling → cutting → drying; fire roasting, charcoal simmering, solar drying	Harvesting: July–August (immature fruits, 5–7 cm in diameter); solar-dried moisture: 10–12%, 3–5 days duration	Heat-induced transformation of polymethoxyflavones and volatile oils	Fire roasting/charcoal simmering enhances aroma and chemical stability; solar drying causes loss of heat-sensitive components and microbial contamination risks	Good controllability of thermal processing; solar drying restricted by weather and unstable quality	[5,5,25,26]
	Modern	Hot air drying, microwave drying, freeze-drying; HAD-ITHC + RF hybrid drying	Hot air drying: 50–60 °C; freeze-drying: −40~−50 °C; optimal moisture transition point: 40%	Volumetric heating, low-temperature sublimation, multi-technology synergy	Microwave drying improves dehydration rate 3–5-fold; freeze-drying retains 18–23% more flavonoids; hybrid drying shortens time by 30.01–37.15% and increases flavonoids by 5.92–9.41%	High efficiency and quality retention for large-scale production; high energy consumption of freeze-drying limits its use to premium products	[27,28,29,30,31]
*Zhishi & Zhiqiao*	Traditional	Zhishi: Harvesting → cleaning → cutting → drying → bran-frying/honey-processing; Zhiqiao: Harvesting → slicing → preliminary drying → bran-frying/honey-processing/fermentation	Zhishi: bran-frying: 120–140 °C, 15% honey solution for honey-processing; Zhiqiao: preliminary drying: 50–60 °C	Thermal decomposition, excipient–herb interaction; deglycosylation of flavonoid glycosides by fermentation	Bran-fried Zhishi regulates qi and invigorates spleen; honey-processed Zhishi relieves cough and tonifies spleen; bran-fried Zhiqiao reduces gastrointestinal irritation; fermentation improves bioavailability	Detoxification and efficacy enhancement for clinical application; rough processing and quality fluctuation	[5,5,32,33,34,35,36]
	Modern	Hot air, vacuum, microwave, infrared, freeze-drying; fresh-cut slice drying	Comparison of drying kinetics and component retention	Selective enrichment of terpenoids, phenolics and flavonoids under different heating modes	Microwave drying optimally retains total phenols and flavonoids; fresh-cut drying achieves high efficiency and high contents of marker compounds	Clear quality indicators for standardization; insufficient research on deep processing modification	[37,38]
*Juhe*	Traditional	Impurity removal → dehydration → stir-frying/salt-processing	Dried moisture: 10–12%; stir-frying: 200–250 °C for 5–60 min	High-temperature conversion of nomilin to obacunone; ionic regulation via salt-processing	Salt-processing strengthens analgesic and anti-inflammatory activities and nitrosamine inhibition; limonoids decrease in content	Simple procedures and wide clinical application; batch-to-batch variation in chemical composition	[5,39,40,41,42]
	Modern	Efficiency improvement, quality standardization, enhanced bioavailability of bioactive components	—	Processing mechanisms referenced from analogous medicinal materials	Improved extraction and absorption efficiency of active constituents	Clear research orientation; lack of systematic technical optimization	—

**Table 3:** Changes in different *Citrus* MEHHs during processing.

Material	Main Processing Factors	Core Chemical Changes	Key Mechanisms	Bioactivity & Quality
*Chenpi*	Aging, co-fermentation, stir-frying, drying/storage	Hesperidin ↓, hesperetin & PMFs ↑; free flavonoids ↑ 48.12% after fermentation; stir-frying raises limonene/γ-terpinene; bound phenolics ↑	Xeromyces drives PMF enrichment; heat/microbes modify flavonoids	Antioxidant activity ↑; stir-frying enhances multiple pharmacological effects; flavor shifts to woody notes
*Huajuhong*	Drying modes, microwave treatment, aging	Naringin/rhoifolin fluctuate with microwave intensity; total flavonoids ↑ under alternating RH drying; naringin crystallizes to form white frost	Oil gland rupture induces crystallization; flavonoid biosynthesis dominates aging	White frost improves anti-inflammation; hyperspectral imaging enables rapid aging identification
*Zhishi*	Bran-frying, scalding	Glycosylated flavonoids ↓; nobiletin & tangeretin ↑ after bran-frying; 22 differential metabolites screened	High-temperature cleavage of glycosidic bonds; thermal oxidation	Bran-frying alleviates strong medicinal property; provides processing markers
*Zhiqiao*	Stir-frying, salt/vinegar-processing, fermentation	Total flavonoids/coumarins ↑; flavonoid glycosides ↓, aglycones ↑ post-fermentation	Heat, salt, acid and microbes regulate deglycosylation	Antioxidant, anti-inflammatory and gastrointestinal-regulating activities ↑; optimizes intestinal microflora
*Juhe*	Salt-processing, stir-frying, storage	Nomilin ↓, obacunone ↑ (thermal conversion); hesperidin bioaccessibility stable	High-temperature conversion of limonoids; storage reshapes volatiles	Obacunone shows stronger anti-inflammation; salt-processing boosts antioxidant capacity

## Data Availability

No new data were created or analyzed in this study. Data sharing is not applicable to this article.
